# A Method to Accurately Estimate the Muscular Torques of Human Wearing Exoskeletons by Torque Sensors

**DOI:** 10.3390/s150408337

**Published:** 2015-04-09

**Authors:** Beomsoo Hwang, Doyoung Jeon

**Affiliations:** Department of Mechanical Engineering, Sogang University, Seoul 121-742, Korea; E-Mail: bshwang@sogang.ac.kr

**Keywords:** exoskeletal robot, human-robot interaction, muscular torque, joint torque sensor

## Abstract

In exoskeletal robots, the quantification of the user’s muscular effort is important to recognize the user’s motion intentions and evaluate motor abilities. In this paper, we attempt to estimate users’ muscular efforts accurately using joint torque sensor which contains the measurements of dynamic effect of human body such as the inertial, Coriolis, and gravitational torques as well as torque by active muscular effort. It is important to extract the dynamic effects of the user’s limb accurately from the measured torque. The user’s limb dynamics are formulated and a convenient method of identifying user-specific parameters is suggested for estimating the user’s muscular torque in robotic exoskeletons. Experiments were carried out on a wheelchair-integrated lower limb exoskeleton, EXOwheel, which was equipped with torque sensors in the hip and knee joints. The proposed methods were evaluated by 10 healthy participants during body weight-supported gait training. The experimental results show that the torque sensors are to estimate the muscular torque accurately in cases of relaxed and activated muscle conditions.

## 1. Introduction

In recent years, there has been increasing interest in using robotic devices to assist in the rehabilitative training of people with motion impairments. Most of the initially developed rehabilitation robots only provide passive-mode training, which moves the user’s limbs along a predefined fixed trajectory. In recent years, many researchers have insisted that robotic assistance should be adaptive according to the user’s contribution for more effective and optimal training [1,2]. In this robotic training paradigm, the quantification of the user’s muscular effort is important to make the robot’s behavior adaptive and to inform the user of their contribution to the training [1]. For example, in rehabilitative training for neurological disorders (e.g., after stroke or spinal cord injury), the patient’s motor performance can be measured and evaluated by the muscular effort estimation.

There are two widely used methods for quantifying the user’s muscular effort in a rehabilitation robot: by measuring electromyography (EMG) using surface electrodes attached to the user’s skin; and by estimating muscular torque based on inverse-dynamics analysis. Although measuring EMG signals has advantages in terms of detecting user intentions with accurate timing, it has practical limitations. For example, the attachment of the electrodes is time-consuming, and complex signal processing is required [3,4]. This paper considers inverse-dynamics-based muscular torque estimation for practical use.

The muscular torque of the human user can be estimated by measuring the applied external torque at each joint of the exoskeletal robot, and by removing the inertial, Coriolis, and gravitational torques of the user’s limb (referred to as “passive torque” throughout the paper to distinguish it from the torque generated by muscle). Computation of the passive torque requires accurate estimates of anthropometric and inertial characteristics of the limb segment, such as mass, center of mass location, and moment of inertia (often referred to as body segment inertial parameters; BSIPs). In inverse-dynamics analyses of human movement, BSIPs are typically estimated from anthropometric models [1,5,6,7]. Although such anthropometric data provide simple solutions for the researcher, they may not match that of the actual user because they cannot provide comprehensive solutions for variations in the gender, race, age, and body type of users [8,9].

In this paper, we present a method for estimating the user’s muscular torque using joint torque sensors and its implementation in an actual exoskeletal robot. In particular, we focus on the identification of user-specific inertial parameters rather than using typical anthropometric models. This approach is important because the isolation of active muscular effort from joint torque measurements critically relies on the accuracy of the dynamic model of the user’s limb. The wheelchair-integrated lower limb exoskeleton robot EXOwheel was used as a test bed, and 10 subjects participated in the experiments. This paper provides a mathematical formulation of the joint torque resulting from the user in the exoskeleton and experimental procedures for identifying user-specific parameters. The performance of the proposed method was verified by experiments on body-weight-supported gait training.

## 2. Mathematical Formulation

**Figure 1:**
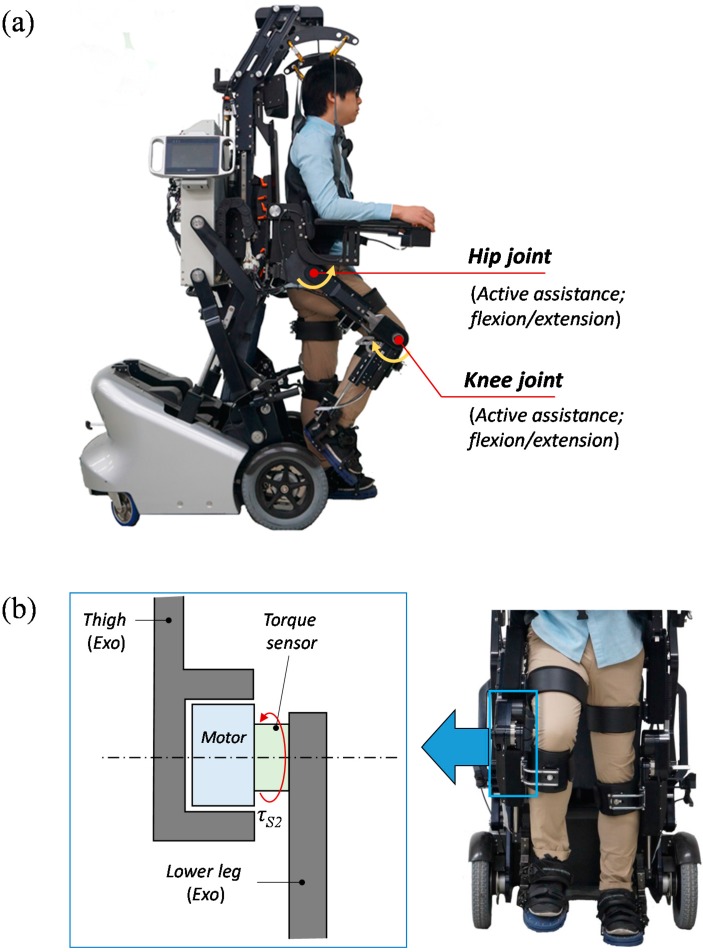
(**a**) Prototype of the EXOwheel robot; (**b**) Schematic diagram of the exoskeleton knee joint in the frontal plane.

Experiments were performed with an EXOwheel robot [10], shown in Figure 1a. The EXOwheel is designed to support exercise and rehabilitative training in the daily lives of individuals with disabilities. The exoskeleton provides assistive joint torques via electric motors in the sagittal plane at the hip and knee joints. The user is connected to the exoskeleton through three attachment points: the thigh, shank, and foot. The length of the thigh and shank in the exoskeleton can be manually adjusted to fit the user’s leg length. Figure 1b shows a schematic diagram of an exoskeleton joint. The exoskeleton is equipped at each joint with an encoder for the motor’s position and a sensor for the joint torque which is located between the motor and link (“torque sensor” in Figure 1b). The exoskeleton’s hip and knee joints have the same configuration. The technical specifications of the torque sensor are: sensing range: ±120 Nm, resolution: 0.015 Nm, non-linearity: 0.03% full scale and repeatability: 0.02% full scale.

**Figure 2:**
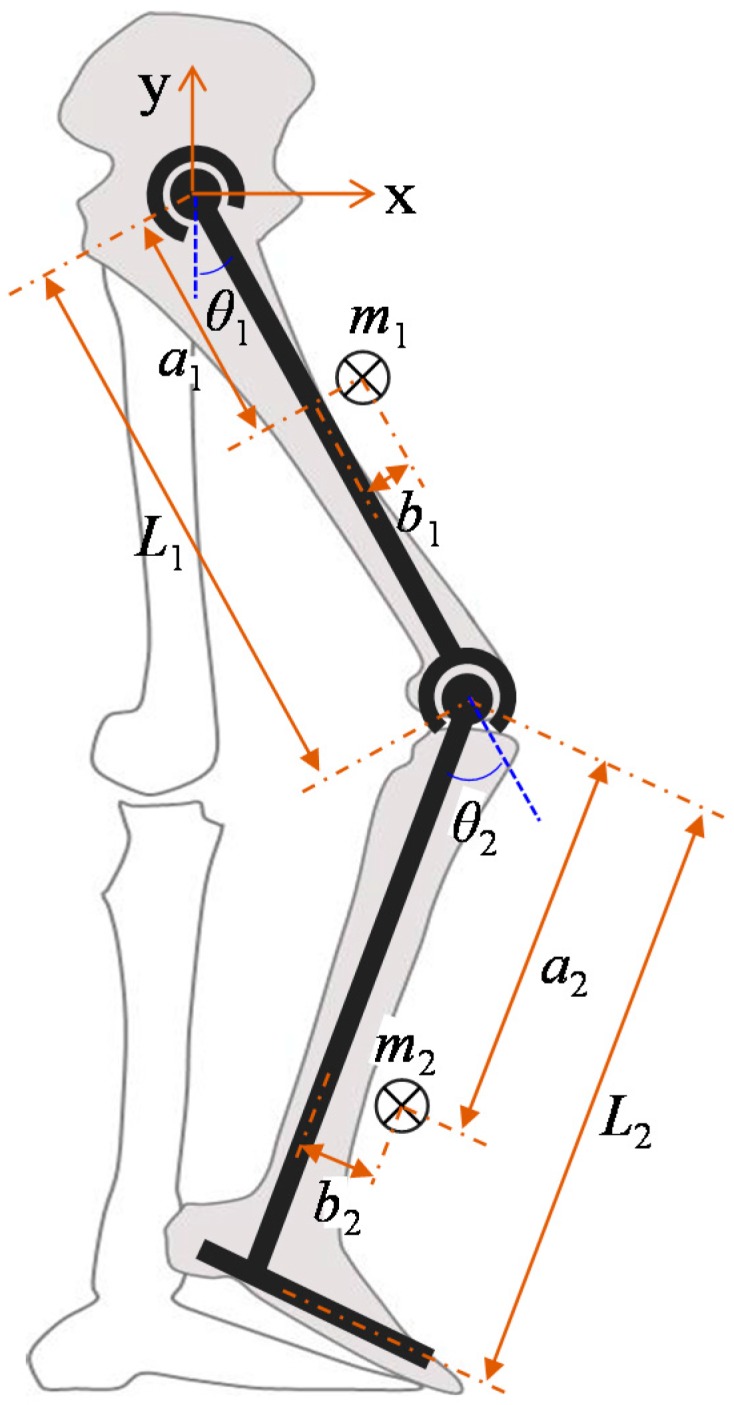
Schematic diagram of the link-segment model of a human lower limb wearing an exoskeleton.

For a human lower extremity wearing an exoskeletal robot, we considered a two-segmental model in the sagittal plane as illustrated in Figure 2. Several assumptions are made to simplify the calculations:

(1) The human leg consists of rigid segments, and each segment is connected with a fixed hinge joint.
(2) The human leg is rigidly linked to the exoskeletal robot, and both systems have the same kinematics.
(3) The model only considers motion in the sagittal plane.
(4) The shank and foot are treated as one rigid segment (*i.e.*, lower leg).

The model consists of two rigid segments (thigh and lower leg) and two pin joints (hip and knee). Each segment of the model is defined by five parameters: length (*L*), mass (*m*), position of center of mass in the directions parallel and perpendicular to the link (*a* and *b*, respectively), and moment of inertia (*I_z_*). *θ* is the joint angle, and positive values of *θ* represent counter-clockwise rotation. Subscript 1 refers to variables of the thigh segment and hip joint, whereas subscript 2 refers to the lower leg segment and knee joint.

The equation of motion for the human lower limb model is expressed as [11]:

(1) $$M_{\text{H}}(\theta )\ddot{\theta }+V_{H}(\theta ,\dot{\theta })+G_{H}(\theta )+P(\theta )=\tau_{M}+\tau_{EXT}$$

where $\theta ,\dot{\theta },\ddot{\theta }\in {ℜ}^{2}$ are the vector of the joint angle, angular velocity, and angular acceleration, respectively. $M_{\text{H}}(\theta )\in {ℜ}^{2\times 2}$ is the symmetric positive definite inertial matrix of the human limb, $V_{H}(\theta ,\dot{\theta })\in {ℜ}^{2}$ is the vector of the centrifugal and Coriolis torques of the human limb, $G_{H}(\theta )\in {ℜ}^{2}$ is the vector of gravitational torques of the human limb, $P(\theta )\in {ℜ}^{2}$ is the vector of passive elastic torques of the human limb, $\tau_{M}\in {ℜ}^{2}$ is the vector of muscular torques, and $\tau_{EXT}\in {ℜ}^{2}$ is the vector of external torques from the environment.

As shown in Equation (1), there are two types of user torque that are applied to the exoskeleton: “passive” torque (motion-dependent torque; *i.e.*, $M_{\text{H}}(\theta )\ddot{\theta }+V_{H}(\theta ,\dot{\theta })+G_{H}(\theta )+P(\theta )$) and “active” torque (muscular torque; *i.e.*, ***τ****_M_*). The external torque, ***τ****_EXT_*, is the applied torque from the environment. In the EXOwheel, the external torque can be expressed by subtracting the torque needed to move the exoskeleton from the torque generated by the robot’s actuator:

(2) $$\tau_{EXT}=\tau_{R}-\left(M_{\text{R}}(\theta )\ddot{\theta }+V_{R}(\theta ,\dot{\theta })+G_{R}(\theta )\right)$$

where $\tau_{R}\in {ℜ}^{2}$ is the vector of the actuator torque applied at the joint, $M_{\text{R}}(\theta )\in {ℜ}^{2\times 2}$ is the symmetric positive definite inertial matrix of the exoskeleton, $V_{R}(\theta ,\dot{\theta })\in {ℜ}^{2}$ is the vector of Coriolis and centrifugal torques of the exoskeleton, and $G_{R}(\theta )\in {ℜ}^{2}$ is the vector of gravitational torques of the exoskeleton. Substituting Equation (2) into Equation (1) yields the following equation of motion for the combined human-exoskeleton system:

(3) $$M_{\text{HR}}(\theta )\ddot{\theta }+V_{HR}(\theta ,\dot{\theta })+G_{HR}(\theta )+P(\theta )=\tau_{M}+\tau_{R}$$

where the index *HR* represents the combined human-exoskeleton system, *i.e.*, **M_HR_** = **M_H_** + **M_R_**, ***V_HR_*** = ***V_H_*** + ***V_R_***, ***G_HR_*** = ***G_H_*** + ***G_R_***.

In Equation (3), **M**_HR_, ***V****_HR_*, and ***G****_HR_* are characterized by the segment inertial parameters: the mass (*m_i_*), the moment of inertia (*I*_z*i*_), and two elements of the center of mass location (*a_i_* and *b_i_*). For the least-squares identification, the unknown parameters *J*, *X*, and *Y* are defined in their linear combinations as follows:

(4) $$\begin{array}{c} J_{1}=I_{z1}+I_{z2}+m_{1}\left(a_{1}^{2}+b_{1}^{2}\right)+m_{2}\left(a_{2}^{2}+b_{2}^{2}\right)+m_{2}L_{1}^{2},\;J_{2}=I_{z2}+m_{2}\left(a_{2}^{2}+b_{2}^{2}\right)\\ X_{1}=m_{1}a_{1}+m_{2}L_{1},\;X_{2}=m_{2}a_{2}\\ Y_{1}=m_{1}b_{1},\;Y_{2}=m_{2}b_{2} \end{array}$$

Indexes *H*, *R*, and *HR* are omitted in Equation (4) for brevity. Then, the equations of **M**_HR_, ***V****_HR_*, and ***G****_HR_* can be written in terms of *J*, *X*, and *Y*:

(5) $$\begin{array}{c} M_{\text{HR}}(\theta )=\left[\begin{array}{cc} M_{\text{HR,11}} & M_{\text{HR,12}}\\ M_{\text{HR,21}} & M_{\text{HR,22}} \end{array}\right]\\ M_{\text{HR,11}}=J_{HR1}+2L_{1}\left(X_{HR2}\cos\theta_{2}-Y_{HR2}\sin\theta_{2}\right)\\ M_{\text{HR,12}}=M_{\text{HR,21}}=J_{HR2}+L_{1}\left(X_{HR2}\cos\theta_{2}-Y_{HR2}\sin\theta_{2}\right)\\ M_{HR,22}=J_{HR2} \end{array}$$

(6) $$\begin{array}{c} V_{HR}(\theta ,\dot{\theta })=\left[\begin{array}{c} \;V_{HR,1}\\ V_{HR,2} \end{array}\right]\\ V_{HR\text{,1}}=-L_{1}\left(X_{HR2}\sin\theta_{2}+Y_{HR2}\cos\theta_{2}\right)({\dot{\theta }}_{2}^{2}+2{\dot{\theta }}_{1}{\dot{\theta }}_{2})\\ V_{HR,2}=L_{1}\left(X_{HR2}\sin\theta_{2}+Y_{HR2}\cos\theta_{2}\right){\dot{\theta }}_{1}^{2} \end{array}$$

(7) $$\begin{array}{c} G_{HR}(\theta )=\left[\begin{array}{c} \;G_{HR,1}\\ G_{HR,2} \end{array}\right]\\ G_{HR,1}=g(X_{HR1}\sin\theta_{1}+Y_{HR1}\cos\theta_{1}+X_{HR2}\sin\theta_{12}+Y_{HR2}\cos\theta_{12})\\ G_{HR,2}=g\left(X_{HR2}\sin\theta_{12}+Y_{HR2}\cos\theta_{12}\right) \end{array}$$

where *g* is gravitational acceleration, *θ*_12_ = *θ*_1_ + *θ*_2_, *J*_HR_ = *J*_H_ + *J*_R_, *X*_HR_ = *X*_H_ + *X*_R_ , and *Y*_HR_ = *Y*_H_ + *Y*_R_.

From the location of the torque sensor (between the actuator and link; see Figure 1b), the measured torque can be described as:

(8) $$\begin{array}{l} \tau_{S}=-\tau_{R}\\ \;=\tau_{M}-\left(M_{\text{HR}}(\theta )\ddot{\theta }+V_{HR}(\theta ,\dot{\theta })+G_{HR}(\theta )+\text{P}(\theta )\right) \end{array}$$

where ***τ****_S_* is the torque measured by the exoskeleton’s torque sensor. The active muscular torque of the human user can be estimated from the measured torque ***τ****_S_* as follows:

(9) $${\hat{\tau }}_{M}=\tau_{S}-{\hat{\tau }}_{PAS}$$

where

(10) $${\hat{\tau }}_{PAS}=-\left({\hat{M}}_{\text{HR}}(\theta )\ddot{\theta }+{\hat{V}}_{HR}(\theta ,\dot{\theta })\text{+}{\hat{G}}_{HR}(\theta )+\hat{P}(\theta )\right)$$

and “hats” are placed on the parameters to denote the estimated values. As shown in Equations (9) and (10), it is important to extract the dynamic effects of the human body and exoskeleton accurately from the measured torque for estimating user’s active muscular torque.

The user’s BISP values in Equation (4) (*m_H,i_*, *a_H,i_*, and *I_zH,i_*) are typically estimated from the literature. Table 1 shows three widely used BSIP estimation models: two models derived from cadaver studies [12,13] and one model derived from the gamma-ray scanning of living subjects [14]. In the table, each segment’s mass (*M*) is described as a ratio of the total weight, whereas the center of mass location (*CM*) and radius of gyration (*RG*) are described as segment length ratios:

(11) $$m_{H,i}=W\times \;M_{i},\;a_{H,i}=L_{i}\times \;CM_{i},\;I_{zH,i}=\;m_{H,i}\times {\left(L_{i}\times RG_{i}\right)}^{2}$$

where *W* is the subject’s weight. The BSIPs estimated from Table 1 are not identical to those of actual users; in this study, the BISPs are not only estimated from the literature but also measured and validated with actual human subjects in the following sections.

**Table 1:** BSIP estimation models.

Studies	*N* ^a^	Method	Thigh			Shank			Foot		
			*M* ^b^	*CM* ^c^	*RG* ^d^	*M*	*CM*	*RG*	*M*	*CM*	*RG*
Dempster [12]	7	Cadaver	9.7	43.3	32.3	4.5	43.3	30.2	1.4	43.8	47.5
Clauser [13]	13	Cadaver	10.3	37.2	-	4.3	37.1	-	1.5	44.9	-
De Leva [14]	100	γ-ray	14.2	45.5	32.9	4.3	40.5	25.5	1.4	55.9	25.7

^a^ *N*: sample size; ^b^ *M*(*%*): percentage of body segment mass relative to total body mass; ^c^ *CM*(*%*): center of mass location as a percentage of the segment length from the proximal end; ^d^ *RG*(*%*): radius of gyration at *CM* for the sagittal axis as a percentage of segment length.

Passive elastic torque, ***P***(***θ***), is a torque generated by the mechanisms of the joint surface, ligaments, and connective tissue. This torque is weak relative to the gravitational and inertial torques, but it becomes significant at the end range of motion [15]. A model for estimating passive elastic torque is based on Riener’s double-exponential equations [16].

(12) $$\begin{array}{c} \;\hat{P}(\theta )=\left[\begin{array}{c} {\hat{P}}_{1}\\ {\hat{P}}_{2} \end{array}\right]\\ {\hat{P}}_{\text{1}}=-\exp(1.47-0.19\theta_{2}-4.29\theta_{1})+\exp(1.34-1.30\theta_{2}+1.75\theta_{1})-8.07\\ {\hat{P}}_{2}=-\exp(1.80-2.02\theta_{2}+1.24\theta_{1})+\exp(-3.97+0.73\theta_{2}-0.0128\theta_{1})\;-\exp(2.22-8.60\theta_{2})+4.82 \end{array}$$

The unit of the angle is radians and the unit of torque is Nm.

## 3. Identification of Body Segment Parameters

### 3.1. Experimental Procedure

To improve the accuracy of the BSIPs, we identified certain parameters from the series of experiments. All experiments were performed on 10 healthy subjects (Table 2). The experimental procedure was approved by the Ethics Committee of Sogang University (approval number: Sogang-IRB-2014-08), and written informed consent was obtained from all participants.

**Table 2:** Subject characteristics.

Subjects	Age (years)	Height (m)	Weight (kg)	BMI ^a^ (kg/m^2^)	*L*_1_ (m)	*L*_2_ (m)
S1	29	1.76	75.2	24.3	0.41	0.53
S2	25	1.78	71.1	22.6	0.42	0.55
S3	24	1.78	74.3	23.5	0.43	0.55
S4	25	1.70	67.6	23.4	0.40	0.52
S5	27	1.78	75.1	23.7	0.42	0.55
S6	24	1.72	65.7	22.2	0.41	0.53
S7	24	1.66	62.8	22.8	0.40	0.52
S8	22	1.72	56.8	19.2	0.40	0.53
S9	23	1.73	65.8	22.0	0.42	0.53
S10	27	1.69	64.6	22.6	0.40	0.51
**Mean**	25.0	1.73	68.0	22.7	0.41	0.53
**SD**	2.2	0.04	5.7	1.3	0.01	0.01

^a^ BMI: Body mass index.

**Figure 3:**
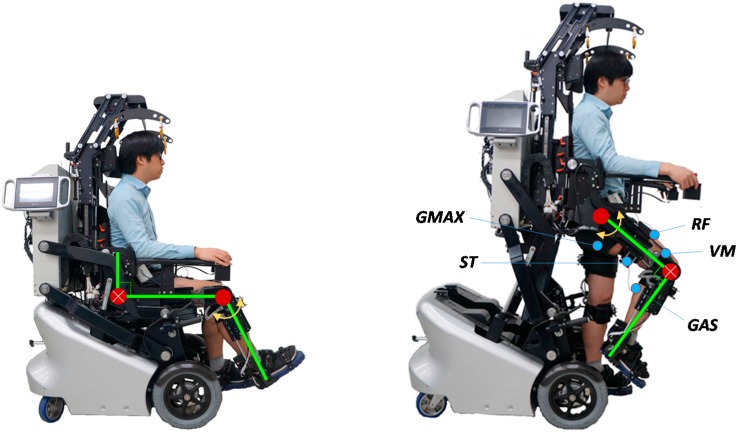
Experimental setup for parameter identification. (**a**) Identification of the lower leg parameters; (**b**) Identification of the thigh parameters.

Figure 3 shows the experimental setup for parameter identification. The subjects were placed in the EXOwheel, and the exoskeleton was connected to their legs. Two experimental configurations were used for parameter identification.

(1) Identification for the lower leg (Figure 3a): The subject sat on the wheelchair seat, which allowed the lower leg to swing. During the experiment, the hip was fixed at 90° flexion, and the knee was moved with a sinusoidal trajectory.
(2) Identification for the thigh (Figure 3b): The subject was in the standing position, which allowed the entire leg to swing. During the experiment, the hip was moved with a sinusoidal trajectory, and the knee was fixed at 90° flexion.

In each experiment, the hip and knee angles were imposed by the exoskeleton, and the subject was asked to fully relax his leg to allow the leg to move passively against the torque imposed by the exoskeleton. Each experiment was carried out five times for 40 s. A conventional proportional derivative (PD) controller was applied at each joint of the exoskeleton to track the desired position. The exciting trajectories were selected as a sum of harmonic sine and cosine functions within the frequency range during the gait training (0.3–1.5 Hz). After the experiments on the subjects were completed, experiments on the exoskeleton only (*i.e.*, exoskeleton not worn by a human subject) were performed in the same configuration to separate the parameters of the exoskeleton and human limb.

### 3.2. Data Analysis and Parameter Identification

The measured joint angles and torques were recorded during the experiment. The angular velocity and acceleration were obtained off-line from the joint angle using a zero-phase digital low-pass filter (Butterworth, second-order, cut-off frequency of 20 Hz). To detect the user’s actual muscular effort during the experiment, EMG data were measured by surface electrodes attached to five major muscles of the right leg in all subjects (Figure 3b): gluteus maximus (GMAX), vastus medialis (VM), rectus femoris (RF), semitendinosus (ST), and gastrocnemius (GAS). The raw EMG signals collected from the electrodes were amplified by a Bagnoli^TM^ 8-channel system (Delsys Inc.) to a gain of 1000 and then band-pass filtered (Butterworth, fourth-order, cut-off frequency of 20–450 Hz). All signals were collected at a sample rate of 1 kHz.

Based on the measured EMG data, muscle activation, *A_ch_*, is classified into two states: “activated” and “inactivated”. The single-threshold method is used as the classification rule:

(13) $$A_{ch}(k)=\{\begin{array}{l} \;1\;(\text{activated}),\text{ if }\chi_{ch}(k)\geq \;Z_{ch}\\ \;0\;(in\text{activated}),\text{ otherwise } \end{array}\;$$

where *χ_ch_*(*k*) is the amplified, band-pass filtered, and full-wave rectified EMG signal of each channel (e.g.*,* GMAX, VL, RF, ST, and GAS) at a discrete time instant *k*. *Z_ch_* is the threshold value, which was set to mean plus three standard deviations (Mean + 3SD) of *χ_ch_* when the muscle is relaxed.

**Figure 4:**
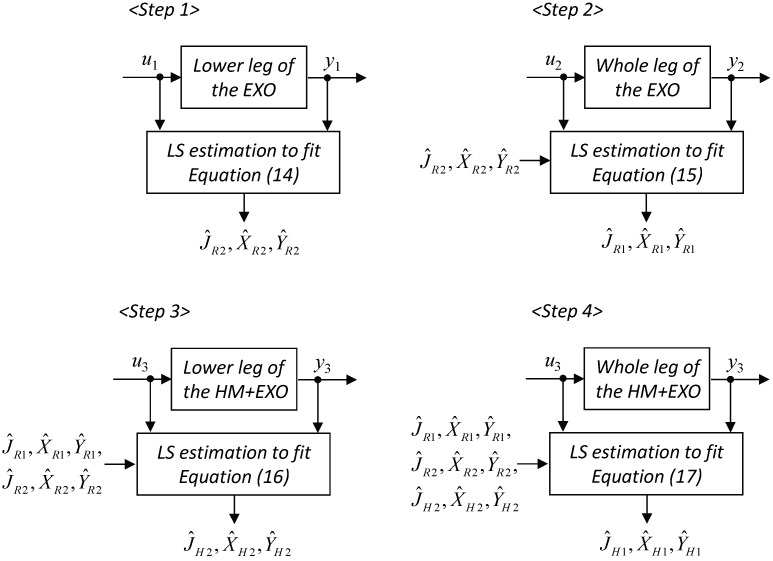
Estimation procedure of the least-squares method. In the figure, *HM* and *EXO* represent the human user and the exoskeleton, respectively.

The parameters *J*, *X*, and *Y* of the exoskeleton and human limb were estimated using the off-line least-squares method. For the human limb parameters, data that satisfy passive conditions (*i.e.*, *A_ch_* = 0 for all EMG channels) were used. The estimation procedures are shown schematically in Figure 4. The procedures were split into two parts: identification of the exoskeleton parameters (Step 1: R2 segment; Step 2: R1 segment) and identification of the human limb parameters with the exoskeleton’s parameters assumed to be known (Step 3: H2 segment; Step 4: H1 segment).

From Equations (3)–(7), the relation between the measured signals, input *u* and output *y*, are expressed as:

Step 1 (R2 segment; ***τM*** = ***0***, *θ*1 = 90°, $\dot{\theta_{1}}$ = $\ddot{\theta_{1}}$ = 0, *J_H,i_* = *X_H,i_* = *Y_H,i_* = 0, *τ_R_*_2_ = *u*_1_, *θ*_2_ = *y*_1_)

(14) $$u_{1}=J_{R2}{\ddot{y}}_{1}+g\left(X_{R2}\cos y_{1}-Y_{R2}\sin y_{1}\right)$$

Step 2 (R1 segment; ***τM*** = ***0***, *θ*2 = − 90 deg, $\dot{\theta_{2}}$ = $\ddot{\theta_{2}}$ = 0, *J_H,i_* = *X_H,i_* = *Y_H,i_* = 0, *τ_R_*_1_ = *u*_2_, *θ*_1_ = *y*_2_)

(15) $$u_{2}=\left(J_{R1}-2L_{1}Y_{R2}\right){\ddot{y}}_{2}+g\left(X_{R1}\sin y_{2}+Y_{R\text{1}}\cos y_{2}-X_{R2}\cos y_{2}+Y_{R2}\sin y_{2}\right)$$

Step 3 (H2 segment; ***τM*** = ***0***, *θ*1 = 90 deg, $\dot{\theta_{1}}$ = $\ddot{\theta_{1}}$ = 0, *τ_R_*_2_ = *u*_3_, *θ*_2_ = *y*_3_)

(16) $$u_{3}=J_{RH2}{\ddot{y}}_{3}+g\left(X_{RH2}\cos y_{3}-Y_{RH2}\sin y_{3}\right)+{\hat{P}}_{2}$$

Step 4 (H1 segment; ***τM*** = ***0***, *θ*2 = − 90 deg, $\dot{\theta_{2}}$ = $\ddot{\theta_{2}}$ = 0, *τ_R_*_1_ = *u*_4_, *θ*_1_ = *y*_4_)

(17) $$u_{4}=\left(J_{RH1}-2L_{1}Y_{RH2}\right){\ddot{y}}_{4}+g\left(X_{RH1}\sin y_{4}+Y_{RH1}\cos y_{4}-X_{RH2}\cos y_{4}+Y_{RH2}\sin y_{4}\right)+{\hat{P}}_{1}$$

Equations (14)–(17) can be written in a linear form as:

(18) $$u_{j}^{*}=w_{j}^{T}\phi_{j},\;j=1,2,3,4,$$

where $u_{j}^{*}\in ℜ$ is the modified input signal, $w_{j}\in {ℜ}^{3}$ is the regression vector as a function of geometry and $y,\dot{y},\ddot{y}$, and $\phi_{j}\in {ℜ}^{3}$ is the vector of the segment parameters. The components of the vectors u_j*_, *w_j_*, and *ϕ_j_* in Equation (18) can be found in Appendix.

When *N* measurements are used, Equation (18) can be represented as:

(19) $$U_{j}={W_{j}}^{T}\phi_{j}$$

where

(20) $$U_{j}=\left[\begin{array}{c} {u_{j}}^{*}(1)\\ {u_{j}}^{*}(2)\\ \vdots \\ {u_{j}}^{*}(N) \end{array}\right],W_{j}^{T}=\left[\begin{array}{c} w_{j}(1)\\ w_{j}(2)\\ \vdots \\ w_{j}(N) \end{array}\right]$$

A least-squares method [17] can be used to estimate the parameters in Equation (23).

(21) $${\hat{\phi }}_{j}={\left({W_{j}}^{T}W_{j}\right)}^{-1}{W_{j}}^{T}U_{j}$$

### 3.3. Identification Results

**Figure 5:**
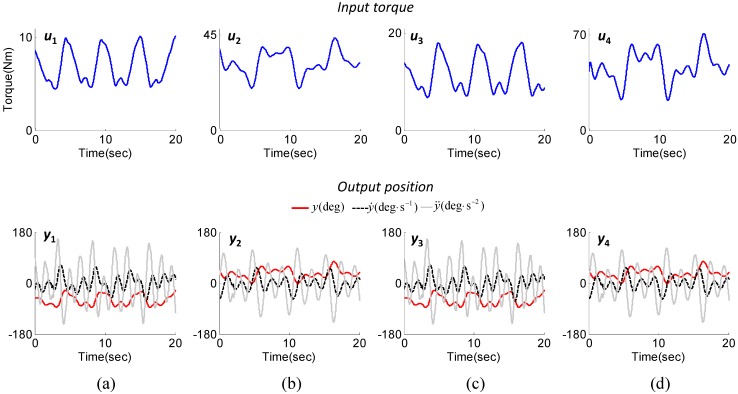
Input (joint torque) and output (joint kinematics) data recorded in the identification experiments for subject 1. (**a**) Step 1 (knee joint data; exoskeleton); (**b**) Step 2 (hip joint data; exoskeleton); (**c**) Step 3 (knee joint data; combined human-exoskeleton system); (**d**) Step 4 (hip joint data; combined human-exoskeleton system).

Figure 5 shows the input and output data recorded in the identification experiments. The exciting trajectories were selected as the sum of harmonic sine and cosine functions considering the joint angle range and frequency in typical gait training in the EXOwheel system. As shown in the second row of Figure 5, the range of the joint angle was 5 to 40 deg for the hip joint and −5 to −70 deg for the knee joint. The range of angular velocity was ±120 deg/s for the hip joint and ±165 deg/s for the knee joint. The kinematic data of the exoskeleton and the combined human-exoskeleton system were identical for each joint (*i.e.*, *y*_1_ = *y*_3_, *y*_2_ = *y*_4_).

Table 3 and 4 represent the results of the parameter identification in the exoskeletal robot and human body, respectively. In Table 3, the identified parameters of exoskeletal robot vary since the link length of the thigh and lower leg in exoskeleton are adjusted to fit the subject’s leg length. The parameters of human body, however, vary significantly though the segment length and weight of individuals are similar (Table 4). For example, S3 and S5 have highly similar weights (*W* = 74.3 kg for S3; *W* = 75.1 kg for S5) and segment lengths (*L*_1_ = 0.43, *L*_2_ = 0.55 for both subjects), but the identified human limb parameters show significant discrepancy between the two subjects (e.g., *X_H_*_1_ = 4.40, *X_H_*_2_ = 1.50 for S3; *X_H_*_1_ = 3.56, *X_H_*_2_ = 1.27 for S5). In particular, parameter *Y_H_*, which represents the distribution of the mass in the direction perpendicular to the link, deviated significantly among all of the subjects regardless of the weight and segment length.

In Table 4, the identified parameters of the human body are compared from that of the two anthropometric models: the De Leva model and the Dempster model. The observed results indicate that the anthropometric data are not sufficient to estimate user-specific parameters. There are two possible reasons for the discrepancies between the parameters identified from the experiments and the anthropometric models. First, normalizing the parameters with respect to weight and segment length alone might cause poor estimation because the identified parameters are sensitive to the body shape of the subject (*i.e.*, spatial distribution of the segment’s mass). Second, the assumptions applied in the anthropometric-model-based estimation (e.g., the radius of gyration of the each segment is constant and the anthropometric segment boundaries coincide with that of the exoskeleton) might increase the discrepancies.

**Table 3:** Parameter identification results for the exoskeletal robot (*J*(kg∙m^2^), *X*(kg∙m), *Y*(kg∙m)).

Subjects	*J_R_*_1_	*J_R_*_2_	*X_R_*_1_	*X_R_*_2_	*Y_R_*_1_	*Y_R_*_2_
S1	3.02	0.74	3.85	1.74	0.15	0.16
S2	3.13	0.77	3.93	1.77	0.15	0.17
S3	3.21	0.77	3.99	1.78	0.12	0.18
S4	2.86	0.71	3.75	1.70	0.15	0.19
S5	3.14	0.77	3.93	1.78	0.13	0.17
S6	3.01	0.73	3.85	1.73	0.17	0.18
S7	2.87	0.71	3.74	1.69	0.13	0.19
S8	2.86	0.74	3.75	1.73	0.13	0.16
S9	3.13	0.74	3.93	1.74	0.13	0.18
S10	2.86	0.68	3.74	1.64	0.16	0.17
**Mean**	3.01	0.74	3.85	1.73	0.14	0.18
**SD**	0.13	0.03	0.10	0.04	0.02	0.01

**Table 4:** Parameter identification results for the human body (*J*(kg∙m^2^), *X*(kg∙m), *Y*(kg∙m)).

Sub-jects	Identified						De Leva				Dempster			
	*J_H_*_1_	*J_H_*_2_	*X_H_*_1_	*X_H_*_2_	*Y_H_*_1_	*Y_H_*_2_	*J_H_*_1_	*J_H_*_2_	*X_H_*_1_	*X_H_*_2_	*J_H_*_1_	*J_H_*_2_	*X_H_*_1_	*X_H_*_2_
S1	1.69	0.37	4.54	1.20	−0.16	0.42	1.48	0.42	3.55	1.16	1.71	0.70	3.21	1.39
S2	1.41	0.50	4.19	1.18	−0.29	0.37	1.48	0.43	3.46	1.14	1.71	0.72	3.13	1.38
S3	1.50	0.40	4.40	1.50	−0.50	0.42	1.61	0.45	3.67	1.19	1.85	0.74	3.32	1.43
S4	1.35	0.36	3.89	1.02	−0.45	0.39	1.27	0.37	3.11	1.02	1.46	0.60	2.81	1.23
S5	1.42	0.48	3.56	1.27	−0.18	0.36	1.55	0.45	3.63	1.20	1.79	0.75	3.28	1.45
S6	1.62	0.33	4.36	0.99	−0.47	0.30	1.29	0.36	3.10	1.00	1.49	0.60	2.80	1.21
S7	1.39	0.46	3.55	0.96	−0.21	0.23	1.18	0.34	2.89	0.95	1.36	0.56	2.61	1.14
S8	1.14	0.42	3.44	1.11	−0.48	0.43	1.06	0.32	2.61	0.87	1.23	0.53	2.36	1.05
S9	1.37	0.41	3.71	1.08	−0.10	0.21	1.36	0.37	3.18	1.01	1.57	0.61	2.87	1.22
S10	1.39	0.37	3.22	1.07	−0.29	0.34	1.21	0.34	2.97	0.96	1.40	0.55	2.69	1.15
**Mean**	1.43	0.41	3.89	1.14	−0.31	0.35	1.34	0.38	3.21	1.05	1.55	0.63	2.90	1.26
**SD**	0.15	0.05	0.44	0.15	0.14	0.07	0.17	0.05	0.33	0.11	0.19	0.08	0.30	0.13

### 3.4. Validation of the Identification Results

The quality of the identified parameters was evaluated in body-weight supported gait training. During the experiment, the exoskeleton was controlled to move along a given trajectory while the subject was asked to fully relax his leg muscles. The trajectories of the hip and knee joint angles were determined from the physiological gait pattern [18], and the gait speed was selected to be 2 km/h (typical range of gait speed in rehabilitation training; approximate stride period of 2 s). During the training, the subject’s body weight was fully supported by the EXOwheel’s electrical lifter for no ground contact. The experiment was carried out for 10 stride cycles on each subject.

**Figure 6:**
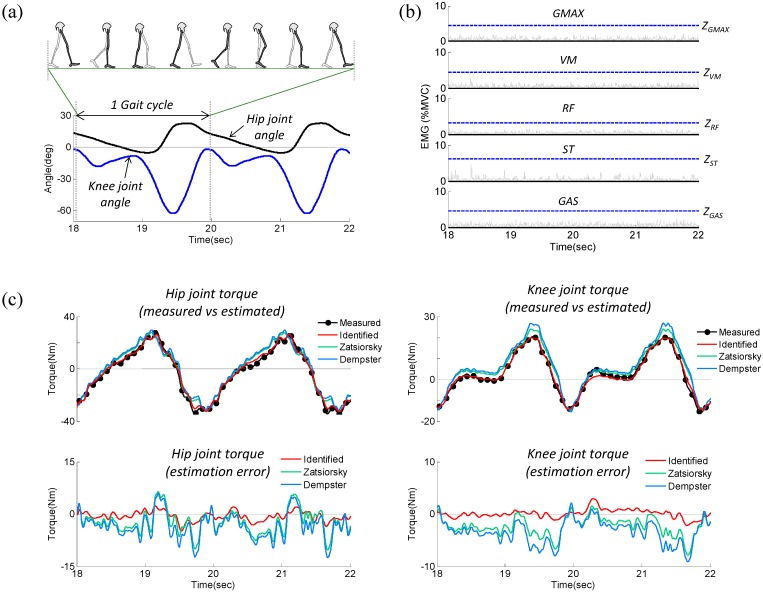
Representative experimental results (subject 1) used to validate the identified parameters while the subject was relaxed. (**a**) Measured joint angle; (**b**) Measured EMG; (**c**) Comparison between measured and estimated torque.

As the subject’s leg moves passively, the measured joint torque only contains motion-dependent “passive” torque (*i.e.*, ${\hat{\tau }}_{PAS}$ in Equation (10)). In the relaxed muscle condition, the estimated torque ${\hat{\tau }}_{PAS}$ should coincide with the measured torque $\tau_{S}$ if the identified parameters are accurate. Figure 6 shows the results of the validation experiment for subject 1, which include measured kinematics (Figure 6a), measured EMG data (Figure 6b), and a comparison between measured and estimated torque (Figure 6c). Figure 6b shows that all of the muscles were in the “inactivate” state, which indicates the subject was fully relaxed throughout the experiment. As shown in Figure 6c, for both the hip and knee joints, there was good agreement between the estimated and measured torques, validating the accuracy of the identified parameters.

The performance of the identified model was quantitatively evaluated by inspecting the root-mean-square (RMS) error of the torque differences between the estimated and measured torque:

(22) $$RMSE=\sqrt{\frac{\sum_{k=1}^{N}{\left(\tau_{S}(k)-{\hat{\tau }}_{PAS}(k)\right)}^{2}}{N}}$$

In calculating the RMS error, the estimated torque ${\hat{\tau }}_{PAS}$ was calculated using the three different parameter sets: experimentally identified parameters and the parameters estimated from the De Leva and Dempster models. According to the results shown in Table 5 for all subjects, the RMS errors range 1.5–2.2 Nm for the hip joint and 0.7–1.2 Nm for the knee joint when torques are computed using the experimentally identified parameters. In contrast, large RMS errors are observed when using the parameters estimated from the De Leva model (3.3–6.5 Nm for the hip joint; 2.0–3.9 Nm for the knee joint) and the Dempster model (2.8–7.3 Nm for the hip joint; 2.3–4.5 Nm for the knee joint). These results demonstrate that the experimentally identified parameter set is acceptable and significantly increases the accuracy of the inverse-dynamics analysis relative to the parameter set estimated from typical anthropometric models.

**Table 5:** RMS error for the estimation of the measured torque in passive-mode gait training.

Subjects	Hip Joint (Nm)			Knee Joint (Nm)		
	Identified	De Leva	Dempster	Identified	De Leva	Dempster
S1	2.1	3.8	4.9	1.2	2.5	3.8
S2	1.5	4.3	2.8	0.9	3.9	2.3
S3	1.7	6.5	7.3	0.7	2.0	4.4
S4	2.0	4.9	5.4	0.7	2.3	4.5
S5	1.5	3.3	2.9	1.1	2.6	4.2
S6	1.9	6.0	6.6	1.0	2.4	4.2
S7	1.7	3.9	3.8	0.9	2.5	4.1
S8	1.7	5.7	6.3	1.2	3.7	3.1
S9	2.2	4.6	4.9	0.8	2.7	3.5
S10	1.8	6.2	6.5	0.8	3.0	3.2
**Mean**	1.8	4.9	5.1	0.9	2.8	3.6
**SD**	0.2	1.1	1.6	0.2	0.6	0.7

## 4. Experimental Validation of Active Muscular Torque Estimation

**Figure 7:**
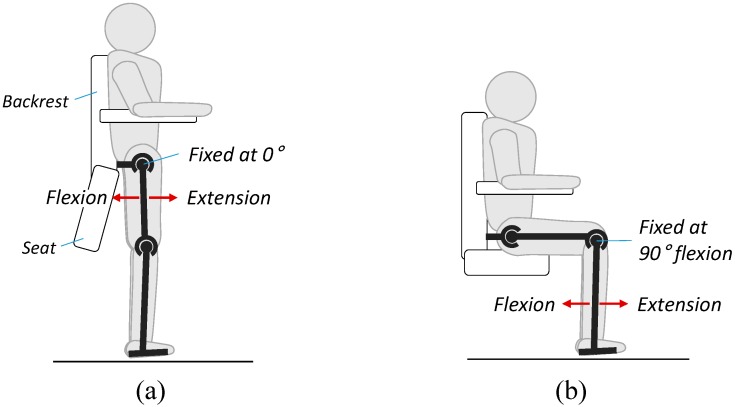
Schematic representation of the isometric calibration procedure. (**a**) Isometric hip flexion and extension; (**b**) Isometric knee flexion extension.

As indicated in Equation (9), the reliability of the estimated active muscular torque ${\hat{\tau }}_{M}$ is guaranteed if the passive torque ${\hat{\tau }}_{PAS}$ is accurately removed from the measured torque $\tau_{S}$. Although the accuracy of the estimated passive torque was analyzed in the previous section, it remains unclear whether the amplitude of the estimation error is sufficiently small to recognize a user’s muscular effort. To validate the estimation of active muscular torque in Equation (9), we investigated the correlation between the estimated muscular torque and EMG data. Prior to the experiment on gait training, the relationship between the EMG data and joint torque was established with isometric contractions. Figure 7 shows the experimental setup for the isometric calibration procedures. The subjects were placed in the EXOwheel, and the exoskeleton was connected to their legs. The subject stood upright during the isometric hip flexion and extension (Figure 7a), whereas the subject sat on the wheelchair seat during isometric knee flexion and extension (Figure 7b). The subjects were asked to perform five to eight isometric flexion and extensions for each joint.

The raw rectified EMG data (*i.e.*, *χ_ch_*) were low-pass filtered (Butterworth, fourth-order, 2 Hz) to obtain linear envelopes. The resulting EMG envelope of each muscle was normalized to the respective muscle’s maximal voluntary contraction (MVC). The subjects were instructed to perform MVCs against manual isometric resistance with specific test positions for each muscle of interest [19]. Three trials were performed with 2 min of resting time between trials; the average of the three highest EMG peaks was used as the MVC value for normalization. 

To calculate muscular torque from the EMG signals, the model reported by Olney and Winter [4] was used in this study. The muscular torques at the hip and knee joint during isometric contraction were calculated from EMG data as follows:

(23) $${\hat{\tau }}_{M1}^{EMG,ISO}(k)=\alpha_{RF,1}\cdot LE_{RF}(k)-\left(\alpha_{GMAX,1}\cdot LE_{GMAX}(k)+\alpha_{ST,1}\cdot LE_{ST}(k)\right)$$

(24) $${\hat{\tau }}_{M2}^{EMG,ISO}(k)=\left(\alpha_{VM,2}\cdot LE_{VM}(k)+\alpha_{RF,2}\cdot LE_{RF}(k)\right)-\left(\alpha_{ST,2}\cdot LE_{ST}(k)+\alpha_{GAS,2}\cdot LE_{GAS}(k)\right)$$

where ${\hat{\tau }}_{M1}^{EMG,ISO}$ and ${\hat{\tau }}_{M2}^{EMG,ISO}$ are, respectively, the muscular torques at the hip and knee joints calculated using EMG under isometric contraction, $LE_{ch}$ is the normalized linear envelope of EMG, and α is a constant relating the amplitude of the $LE_{ch}$ to the joint torque. α is determined by a least-squares curve-fitting procedure as follows:

(25) $$\begin{array}{l} \text{find }\alpha_{i}\\ \text{that minimize }J_{i}^{ISO}={\sum_{k}\left({\hat{\tau }}_{M,i}^{ISO}(k)-{\hat{\tau }}_{M,i}^{EMG,ISO}(k)\right)}^{2},\;i=1,\;2 \end{array}$$

where ***α***1 = {*αRF,*1, *αGMAX,*1, *αST,*1}, ***α***2 = {*αVM,*2, *αRF,*2, *αST,*2, *αGAS,*2}, and ${\hat{\tau }}_{M,i}^{ISO}(k)=\tau_{S,i}(k)$. Note that the measured torque at the joint torque sensor only contains the user’s muscular torque in the experimental setup shown in Figure 7, which indicates that ${\hat{\tau }}_{M,i}^{ISO}$ is reliable during the isometric calibration procedures regardless of the accuracy of the inverse dynamics model. 

**Figure 8:**
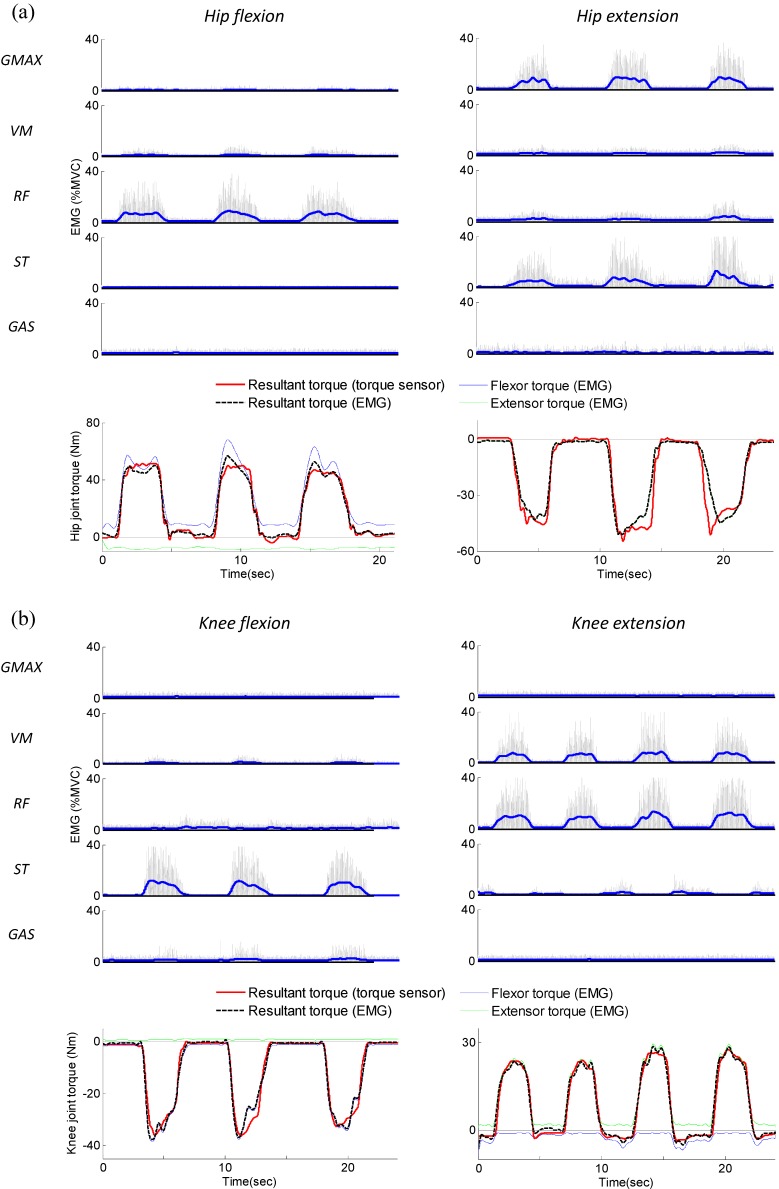
EMG signals and corresponding muscular torques resulting from the isometric calibration procedure (Subject 1). (**a**) Isometric hip flexion and extension; (**b**) Isometric knee flexion and extension.

The optimized values of *α* computed by the least-squares method are (Mean ± SD): ***α***1 = {1.88 ± 0.76, 1.08 ± 0.42, 1.70 ± 0.83}, ***α***2 = {2.53 ± 0.77, 0.89 ± 0.37, 2.91 ± 1.12, 1.51 ± 0.35}. The results of the isometric calibration for the hip and knee joint of subject 1 are presented in Figure 8a,b, respectively. In each figure, a time plots of the EMG signals recorded from GMAX, VM, RF, ST, GAS, and the corresponding muscular torques are presented. In the EMG graphs of Figure 8, thin grey lines represent the raw-rectified EMG signals, and thick blue lines represent EMG linear envelopes. As shown in the torque graphs of Figure 8, good agreement was observed between the muscular torque measured from the torque sensor (solid red line in the figure) and the torque calculated using EMG (dashed black line in the figure). The *R*^2^ values and normalized RMS errors between ${\hat{\tau }}_{M}^{ISO}$ and ${\hat{\tau }}_{M}^{EMG,ISO}$ for all 10 subjects are presented in Table 6. The average *R*^2^ values were high (ranging between 0.951 and 0.976), and the normalized RMS errors were low (ranged between 5.03% and 8.12%) for all isometric contractions; this indicates that the EMG to torque processing model produced accurate estimates of the joint torque.

**Table 6:** Average *R*^2^ and normalized RMS errors between ${\hat{\tau }}_{M}^{ISO}$ and ${\hat{\tau }}_{M}^{EMG,ISO}$ during isometric contraction. Standard deviations are presented in parentheses.

Joint		*R*^2^	% RMSE
Hip	Flexion	0.968 (0.013)	6.24 (1.53)
	Extension	0.951 (0.019)	8.12 (2.37)
Knee	Flexion	0.962 (0.016)	5.96 (1.88)
	Extension	0.976 (0.012)	5.03 (1.61)

After the calibration was completed, the experiment was conducted on gait training. The experimental setup was the same as that used in the model-validation experiment, *i.e.*, body weight supported gait training with pre-defined gait pattern (gait speed: 2 km/h). In this case, the subjects were asked to generate more force than required to follow the pre-defined gait trajectory (*i.e.*, gait with exaggerated flexion/extension of hip and knee joint). The experiment was performed for 10 stride cycles on each subject.

To calculate muscular torque from the EMG signals during dynamic contractions, the model reported by Olney and Winter [4] was used in this study. The muscular torques at the hip and knee joint during dynamic contraction were calculated as follows:

(26) $$\begin{array}{l} {\hat{\tau }}_{M1}^{EMG,DYN}(k)=\alpha_{RF,1}\cdot LE_{RF}(k)\left\{1+\beta_{1}(\theta_{1}(k)-\theta_{1}^{c})-\gamma_{1}{\dot{\theta }}_{1}(k)\right\}\\ \;-\left(\alpha_{GMAX,1}\cdot LE_{GMAX}(k)+\alpha_{ST,1}\cdot LE_{ST}(k)\right)\left\{1-\beta_{1}(\theta_{1}(k)-\theta_{1}^{c})+\gamma_{1}{\dot{\theta }}_{1}(k)\right\} \end{array}$$

(27) $$\begin{array}{l} {\hat{\tau }}_{M2}^{EMG,DYN}(k)=\left(\alpha_{VM,2}\cdot LE_{VM}(k)+\alpha_{RF,2}\cdot LE_{RF}(k)\right)\left\{1+\beta_{2}(\theta_{2}(k)-\theta_{2}^{c})-\gamma_{2}{\dot{\theta }}_{2}(k)\right\}\\ \;-\left(\alpha_{ST,2}\cdot LE_{ST}(k)+\alpha_{GAS,2}\cdot LE_{GAS}(k)\right)\left\{1-\beta_{2}(\theta_{2}(k)-\theta_{2}^{c})+\gamma_{2}{\dot{\theta }}_{2}(k)\right\} \end{array}$$

where ${\hat{\tau }}_{M1}^{EMG,DYN}$ and ${\hat{\tau }}_{M2}^{EMG,DYN}$ are, respectively, the muscular torques at the hip and knee joints calculated using EMG under dynamic contraction. *β* is a constant, in deg−1, depending on the difference between the joint angle θ and the angle $\theta^{c}$ at which the isometric calibration trials were conducted ($\theta_{1}^{c}$ = 0, $\theta_{2}^{c}$ = − 90 deg). *γ* is a constant, in (deg/s)^−1^, accounting for the variations in angular velocities. The optimized values of *β* and *γ* were determined by a least-squares curve-fitting procedure. The optimization problem was formulated as follows:

(28) $$\begin{array}{l} \text{find }\beta_{i},\;\gamma_{i}\\ \text{that minimize }J_{i}^{DYN}={\sum_{k}\left({\hat{\tau }}_{M,i}(k)-{\hat{\tau }}_{M,i}^{EMG,DYN}(k)\right)}^{2},\;i=1,\;2 \end{array}$$

where ${\hat{\tau }}_{M,i}\left(k\right)=\tau_{S,i}\left(k\right)-{\hat{\tau }}_{PAS,i}(k)$.

**Figure 9:**
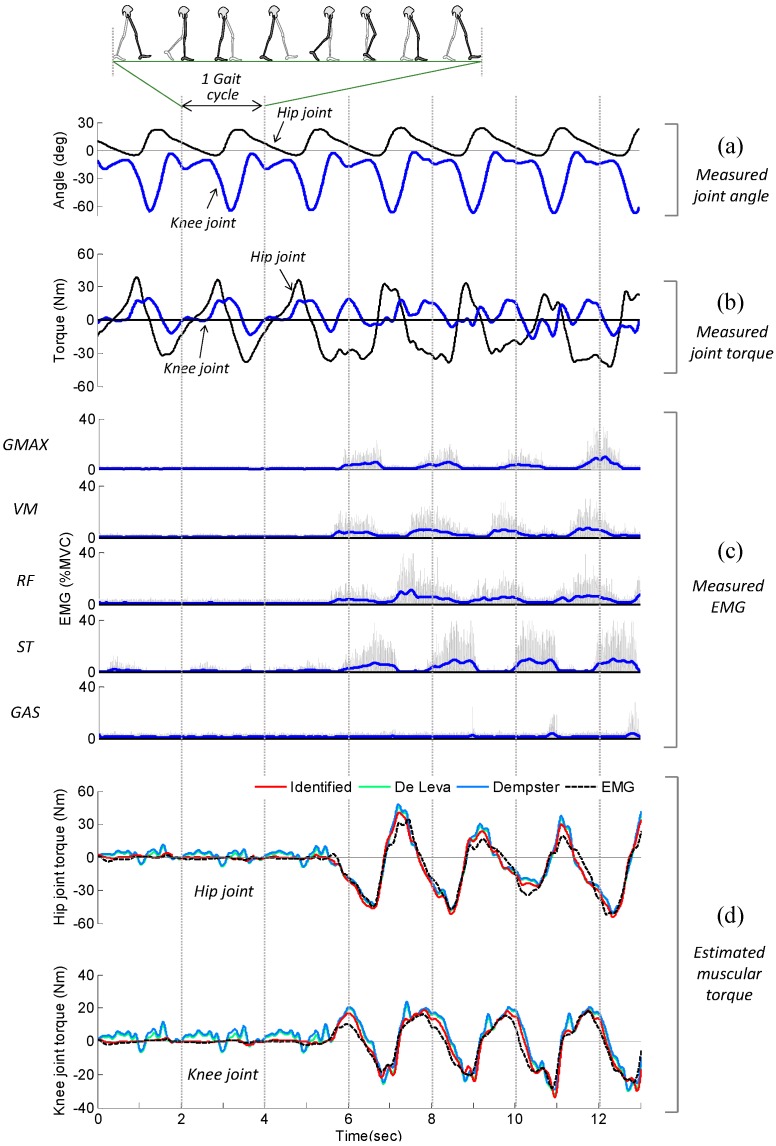
EMG signals and corresponding muscular torques resulting from the experiment on gait training (subject 1). (**a**) Measured joint angle; (**b**) Measured joint torque; (**c**) Measured EMG; (**d**) Estimated muscular torque.

The optimized values of *β* and *γ* computed by the least-squares method are presented in Table 7. The values of *α* were derived from the isometric calibration procedure. Figure 9 shows the results of the gait experiment for subject 1, which include the measured EMG, measured joint angle, measured joint torque, and estimated muscular torque. The subject was fully relaxed up to 5 s, and then generated muscular force with exaggerated flexion/extension of the hip and knee joint. During the periods when the subject was passive (*i.e.*, 0–5 s), the measured torque exhibited a repetitive pattern (Figure 9b). This behavior can be explained by the fact that most of the measured torque was induced by the passive torque, such as the inertial, Coriolis/centrifugal, and gravitational torque of the subject’s limb. It can be observed that the active muscular torque estimated using inverse dynamics (solid red lines in Figure 9d) was close to zero over the period 0–5 s, indicating the passive torque was accurately removed from the measured torque.

**Table 7:** Average values of the optimal coefficients, *R*^2^ and normalized RMS errors for the experiment on gait training. Standard deviations are presented in parentheses.

Model	Joint		Optimal Coefficients (10^−3^)		*R*^2^	% RMSE
			*β*	*γ*		
Identified		Hip	3.27 (1.53)	1.45 (0.57)	0.935 (0.028)	8.74 (2.63)
		Knee	2.44 (1.31)	0.92 (0.35)	0.924 (0.034)	10.26 (3.06)
De Leva		Hip	2.05 (1.27)	0.87 (0.52)	0.884 (0.046)	11.31 (3.83)
		Knee	1.46 (1.05)	0.66 (0.44)	0.877 (0.048)	14.73 (4.05)
Dempster		Hip	2.14 (1.36)	0.84 (0.48)	0.879 (0.042)	11.67 (3.74)
		Knee	1.37 (0.92)	0.63 (0.40)	0.872 (0.051)	15.38 (4.12)

During the periods when the subject was active (*i.e.*, after 5 s), the pattern observed for the measured torque was not repetitive because it featured the active muscular torque in addition to the passive torque (Figure 9b). Figure 9d shows a comparison of the muscular torque estimated using inverse dynamics, ${\hat{\tau }}_{M}$, and the muscular torque calculated using EMG, ${\hat{\tau }}_{M}^{EMG,DYN}$. As expected, good agreement was observed between ${\hat{\tau }}_{M}$ (solid red line in the figure) and ${\hat{\tau }}_{M}^{EMG,DYN}$ (dashed black line in the figure). The *R*^2^ values and normalized RMS errors between ${\hat{\tau }}_{M}$ and ${\hat{\tau }}_{M}^{EMG,DYN}$ for all 10 subjects are presented in Table 7. As seen in Figure 9d and Table 7, the estimated muscular torque and EMG data are closer when using the experimentally identified parameter set than using the parameter set estimated from typical anthropometric models. The average *R*^2^ is 0.935 for the hip joint and 0.924 for the knee joint when using the experimentally identified parameter set and it shows that the estimated muscular torque during gait experiment is highly correlated with the EMG data. This indicates that most of the estimated muscular torque was induced by the subject’s muscle activation (*i.e.*, neural command) and the proposed method can be effectively used to estimate the user’s muscular effort.

## 5. Conclusions

This study presents a method for estimating users’ active muscular torque measured by sensor systems typically used in exoskeletal rehabilitation robots (*i.e.*, encoder and torque sensors). The key step in this method was to accurately identify the inertial parameters of the user’s limb. Parameters experimentally derived from actual users are significantly more accurate than those obtained by widely used anthropometric models. Experimental results on gait training validate the proposed muscular torque estimation method. 

The method proposed in this study is valid only when the user moves in the air with no ground contact because ground reaction force (GRF) cannot be measured with the current EXOWheel system. This method can also be extended to over-ground walking with measurement of the GRF. When the foot is in contact with the ground, however, the accuracy of the GRF measurements will likely become the critical factor in estimating muscular torque rather than the accuracy of the user’s limb inertial parameters. A future study will examine the performance of this method during ground contact.

The advantages of the proposed method can be summarized as follows: (1) no additional sensors for measuring bio-signals are necessary because this method only uses common rehabilitation robot sensors and (2) the method provides user’s muscular effort in terms of joint torque, which is adequate as a feedback signal for the controller in joint space; this method can be used for the shared control or adaptive control of exoskeletons.

## Appendix

The components of the vectors *u_j*_*, *w_j_*, and *ϕ_j_* in Equation (18) are expressed as follows:

(A1) $$u_{1}^{*}=u_{1},\;w_{1}=\left[\begin{array}{c} {\ddot{y}}_{1}\\ g\cos y_{1}\\ g\sin y_{1} \end{array}\right],\;\phi_{1}=\left[\begin{array}{c} J_{R2}\\ X_{R2}\\ Y_{R2} \end{array}\right]$$

(A2) $$u_{2}^{*}=u_{2}+2L_{1}Y_{R2}{\ddot{y}}_{2}-g\left(-X_{R2}\cos y_{2}+Y_{R2}\sin y_{2}\right),\;w_{2}=\left[\begin{array}{c} {\ddot{y}}_{2}\\ g\sin y_{2}\\ g\cos y_{2} \end{array}\right],\;\phi_{2}=\left[\begin{array}{c} J_{R1}\\ X_{R1}\\ Y_{R1} \end{array}\right]$$

(A3) $$u_{3}^{*}=u_{3}-{\hat{P}}_{2}+J_{R2}{\ddot{y}}_{3}-g\left(X_{R2}\cos y_{3}-Y_{R2}\sin y_{3}\right),\;w_{3}=\left[\begin{array}{c} {\ddot{y}}_{3}\\ g\cos y_{3}\\ g\sin y_{3} \end{array}\right],\;\phi_{3}=\left[\begin{array}{c} J_{H2}\\ X_{H2}\\ Y_{H2} \end{array}\right]$$

(A4) $$\begin{array}{l} u_{4}^{*}=u_{4}-{\hat{P}}_{1}-\left(J_{R1}-2L_{1}Y_{RH2}\right){\ddot{y}}_{4}-g\left(X_{R1}\sin y_{4}+Y_{R1}\cos y_{4}-X_{RH2}\cos y_{4}+Y_{RH2}\sin y_{4}\right),\;\\ \;w_{4}=\left[\begin{array}{c} {\ddot{y}}_{4}\\ g\sin y_{4}\\ g\cos y_{4} \end{array}\right],\;\phi_{4}=\left[\begin{array}{c} J_{H1}\\ X_{H1}\\ Y_{H1} \end{array}\right] \end{array}$$
